# A particularly atypical case of atypical flutter in a patient after a Mustard repair for d-transposition of the great arteries

**DOI:** 10.1016/j.hrcr.2025.08.018

**Published:** 2025-08-22

**Authors:** Amandeep Kaur, Michael Malaty, Amardeep Amardeep, Nicholas Collins, Krishnakumar Nair, Nicholas Jackson

**Affiliations:** 1 Department of Cardiology, John Hunter Hospital, New Lambton Heights, New South Wales, Australia; 2 The University of Newcastle, Callaghan, New South Wales, Australia; 3 Toronto General Hospital, Toronto, Ontario, Canada

**Keywords:** Mustard repair, Perimitral flutter, d-Transposition of the great arteries, Congenital heart disease, Atypical atrial flutter ablation

**Key Teaching Points**

• Intra-atrial reentrant tachycardia (IART) is seen in at least 30% of patients after a Mustard/Senning procedure for dextro-transposition of the great arteries.
• The most common IART seen is cavotricuspid isthmus–dependent atrial flutter since the Mustard repair predominantly alters the right atrium and tricuspid annular region.
• Given possible challenges with performing a transbaffle puncture in some situations (such as baffles with calcified prosthetic material), we show how a detailed map of the pulmonary venous atrium can be obtained using a retrograde approach with a multipolar mapping catheter.
• This case demonstrates that perimitral annular flutter is also possible after a Mustard repair and that it can be successfully treated with a mitral isthmus line at 9 o’clock on the isthmus to achieve bidirectional block without affecting the native conduction system.

## Introduction

Despite the fact that peritricuspid annular flutter is common after Mustard repair for dextro-transposition of the great arteries (d-TGA), perimitral annular flutter has not been reported. We report the first case of perimitral annular flutter after Mustard repair in a patient who also had atrioventricular (AV) reentry using a left-sided accessory pathway. Both these arrhythmias were successfully treated with ablation therapy. We discuss the challenges in mapping atrial flutter after Mustard repair, approaches to safely ablate a mitral isthmus line in these patients, and the reasons why perimitral flutter is an uncommon arrhythmia in this setting.

## Case report

A 53-year-old man with a previous atrial switch operation (Mustard repair) for d-TGA presented to hospital with recurrent palpitations and dyspnea. The patient underwent a Mustard repair at age 1 and later had surgical correction for inferior baffle stenosis and pulmonary venous return obstruction at age 22. A dual-chamber permanent pacemaker for sinus node dysfunction was implanted at the time of this surgery. The patient subsequently underwent an electrophysiology study and successful ablation for orthodromic atrioventricular reentrant tachycardia using a left lateral accessory pathway at age 43^1^ and an upgrade to a dual-chamber implantable cardioverter-defibrillator (ICD) at age 47 for primary prevention because of moderately impaired systemic ventricular function.

**Figure 1:**
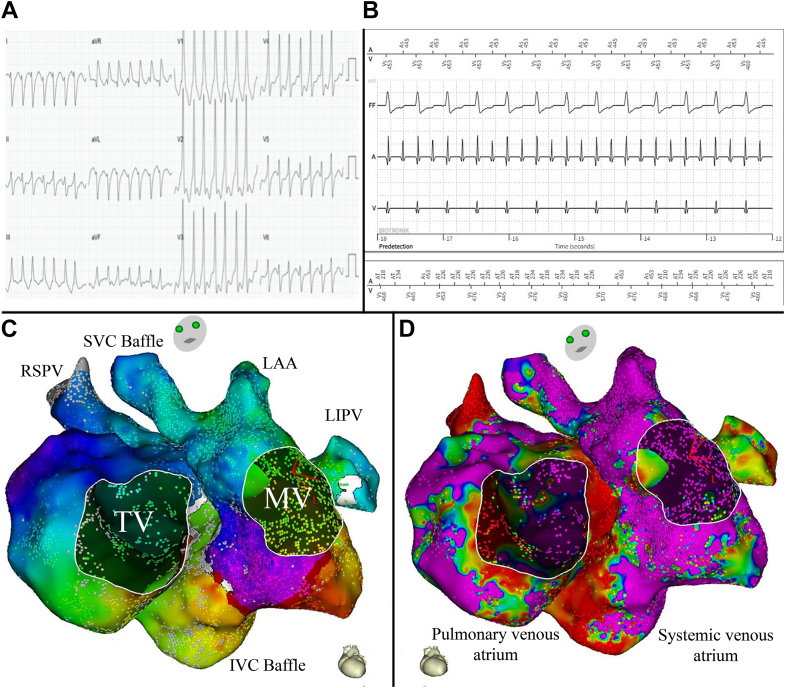
**A:** Twelve-lead electrocardiogram of the patient’s tachycardia. The ventricular rate is 260 beats/min, suggesting 1:1 atrioventricular conduction at this point. The QRS had a right bundle branch block morphology (130 ms) and a right axis deviation (common in dextro-transposition of the great arteries with a Mustard repair), which was identical to the QRS in sinus rhythm. **B:** Electrograms from the interrogation of the patient’s implantable cardioverter-defibrillator. These show a regular ventricular rhythm with what appears to be 2:1 conduction to the ventricle. **C:** An activation map of the systemic and pulmonary venous atria is shown, with the complete cycle length of the tachycardia around the mitral annulus. **D:** A voltage map of the systemic and pulmonary venous atria is shown, which corresponds to the activation map. Low voltage (<0.05 mV; *red*) is seen at the inferior vena cava (IVC) baffle and at the medial aspect of the tricuspid valve (TV). Healthy voltage (>0.5 mV) is shown in *purple*. LAA = left atrial appendage/systemic venous appendage; LIPV = left inferior pulmonary vein; MV = mitral valve; RSPV = right superior pulmonary vein; SVC Baffle = superior vena cava baffle.

At presentation, an electrocardiogram demonstrated a narrow complex tachycardia at 260 beats/min (Figure 1A) and ICD interrogation (Figure 1B) suggested atrial flutter with variable conduction to the ventricle (1:1 on the electrocardiogram and 2:1 on the ICD electrograms). The patient had ongoing episodes of the tachycardia despite maximally tolerated doses of sotalol, and an electrophysiology study was planned.

The electrophysiology study was initially performed under local anesthesia and sedation. Three sheaths were placed into the right common femoral vein (6, 7, and 8 F) and 1 sheath was placed into the right common femoral artery (8 F) under ultrasound guidance. A DecaNav (Johnson & Johnson, MedTech) catheter was placed with the tip in the venous atrial appendage, and a quadrapolar catheter was placed in the subpulmonary, morphologic left ventricle.

There was no evidence of accessory pathway recurrence on anterograde or retrograde testing. Sustained atrial flutter was induced after burst atrial pacing on isoprenaline at 2 μg/min. The atrial cycle length was 200 ms with both 1:1 and 2:1 conduction to the ventricle (consistent with the clinical tachycardia). The tachycardia was hemodynamically tolerated, and activation mapping was performed in the superior and inferior venous baffles and venous atrium using an OctaRay catheter with 2-5-2-5-2 mm spacing (Johnson & Johnson MedTech) as well as in the pulmonary venous atrium via a retrograde aortic approach with the same catheter.

**Figure 2:**
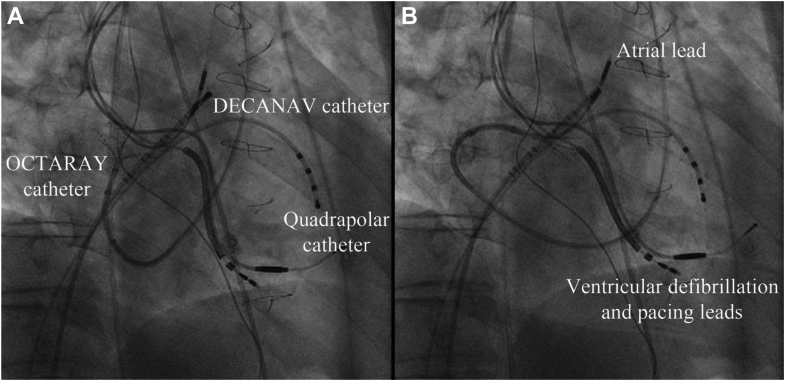
**A:** A fluoroscopy image in the right anterior oblique (RAO) view showing the location of the electrophysiology catheters (labeled) during mapping. The atrial pacing lead is shown in the systemic venous atrial appendage, and an abandoned passive pacing lead and a defibrillation lead are shown in the subpulmonary ventricle. The OctaRay catheter has splines at the ostium of the left inferior pulmonary vein. **B:** A fluoroscopy image in the RAO view showing the same catheters; however, here the OctaRay catheter can be seen flexed back on itself to map the medial aspect of the pulmonary venous atrium.

Activation mapping was consistent with perimitral annular flutter (Figure 1C), and voltage mapping showed low voltage at the inferior baffle and at the medial aspect of the pulmonary venous atrium (Figure 1D). Mapping of the pulmonary venous atrium (PVA) was performed with fluoroscopy in the right anterior oblique view to help maintain the catheter direction posteriorly across the tricuspid valve (Figure 2A and 2B). Using the flexible nature of the OctaRay shaft and splines, the catheter can be systematically advanced and withdrawn, flexed back on itself, and rotated clockwise and counterclockwise to achieve a complete map via the retrograde approach.

**Figure 3:**
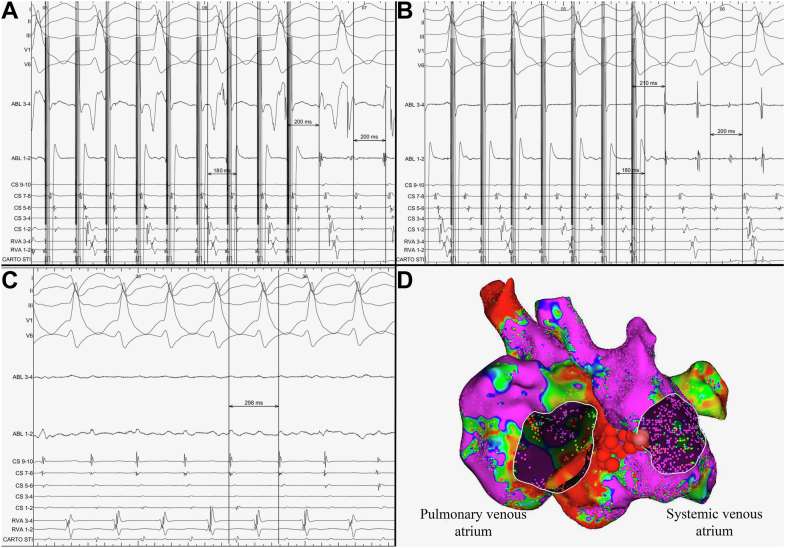
**A:** Five electrocardiogram leads are shown, followed by an ablation catheter at 9 o’clock on the mitral annulus, a DecaNav catheter with the tip in the left atrial appendage, and a catheter labeled right ventricle, which is in the left ventricular apex. Entrainment at 9 o’clock on the mitral annulus with the ablation catheter accelerates tachycardia to the paced cycle length before the resumption of atypical flutter with 2:1 conduction to the ventricle. The PPI − TCL here is 0 ms, confirming that this point is in the circuit. **B:** Entrainment at 5 o’clock on the mitral annulus with a PPI − TCL of 8 ms is shown, also confirming that this site is in the arrhythmia circuit. **C:** Slowing of the atrial cycle length is seen with ablation at 9 o’clock on the mitral annulus, and this leads to resumption of 1:1 conduction to the ventricle. **D:** Ablation dots are shown in *maroon* from 9 o’clock on the mitral annulus back to low voltage at the inferior vena cava baffle posteriorly. This lesion set terminated the tachycardia, and then bidirectional block across this line was shown. PPI − TCL = post pacing interval minus tachycardia cycle length.

Entrainment from 5 o’clock and 9 o’clock on the mitral annulus had a short post pacing interval minus tachycardia cycle length (PPI − TCL) at both these locations (Figure 3A and 3B). Mapping was performed to identify a His potential in both atria, however, one could not be identified. Given that the 9 o’clock position on the mitral isthmus entrained in and that this site allowed for a line to be anchored to low-voltage tissue posteriorly, ablation was performed through 9 o’clock at 40 W (targeting an ablation index of 550) using a ThermoCool ST SF ablation catheter (Johnson & Johnson MedTech). This resulted in slowing (Figure 3C) and then termination of the tachycardia prior to completion of the line (Figure 3D). Bidirectional block was confirmed with pacing and mapping on either side of the line during a 30-minute waiting period. No arrhythmias were inducible with 3 extrastimuli down to refractoriness, and no further arrhythmia recurrence has been seen in 9 months of follow-up.

## Discussion

We present the first case, to our knowledge, of perimitral annular flutter in a patient with a Mustard repair for d-TGA. This was successfully terminated with a radiofrequency ablation line at the medial aspect of the mitral annulus, with no recurrence at 9 months of follow-up.

Atrial flutter or intra-atrial reentrant tachycardia occurs in at least 30% of patients after a Mustard repair for d-TGA.^2^ The combination of surgical incisions, baffles, and atrial dilation from abnormal hemodynamic stress contributes to this high incidence. By far, the most common atrial tachyarrhythmia seen in these patients is cavotricuspid isthmus (CTI)–dependent atrial flutter, observed in up to 75% of patients during the electrophysiology study after an atrial switch procedure for d-TGA.^3^ This likely relates to the fact that the Mustard repair predominantly alters the right atrium and tricuspid annular region with incisions, suturing of the inferior vena cava baffle through the CTI and resultant scarring, combined with the innate predisposition of the CTI to support macroreentrant tachycardia.^4^ In the largest series by Chiriac et al,^3^ CTI-dependent flutter was seen in 75% of patients, right atriotomy–related intra-atrial reentrant tachycardia was seen in 53%, and focal atrial tachycardia was seen in 6% of patients. A number of smaller series examining atrial arrhythmias after atrial switch procedures (totaling 32 patients) also did not report perimitral annular flutter.^5^, ^6^, ^7^

In this case, perimitral flutter was confirmed by observing the complete atrial cycle length only around the mitral annulus and by demonstrating a short PPI − TCL after entrainment from 2 anatomically separate locations on the annulus. In this patient, low-voltage tissue was seen posterior to the mitral annulus (on the medial aspect and in the region of the baffles), which likely contributed to the posterior block necessary for sustained perimitral flutter. This patient was also significantly older than other patients in arrhythmia series' after Mustard repair (53 years vs 38 ± 7 years in Chiriac et al^3^), which may have allowed more time for atriopathy to develop with poorer cell-to-cell electrical coupling and a substrate more conducive to posterior functional block that enables perimitral flutter to initiate and sustain.

Mapping of the PVA was performed using a retrograde approach in order to avoid the need to perform a transbaffle puncture. It is common to perform a transbaffle puncture upfront in patients with atrial tachyarrhythmias after a Mustard repair, as most arrhythmias are located (at least in part) in the PVA.^2^, ^3^, ^4^, ^5^, ^6^, ^7^ Furthermore, given the prevalence of CTI-dependent flutter, the transbaffle approach may allow better contact and stability on the CTI itself. Drawbacks of the transbaffle technique include that the baffle puncture can be time-consuming and difficult to cross (especially through calcified prosthetic material) and even with balloon dilatation the puncture may be tight, which can limit mapping and maneuverability.^8^ We have shown that detailed retrograde mapping is possible with modern catheters (particularly without significant right ventricular dilatation), and this can potentially avoid the need for a routine baffle puncture.

In this case, we chose to perform an ablation line at 9 o’clock on the mitral annulus. This point was demonstrated to be in the circuit on entrainment; it enabled good contact force and stability; and it provided the shortest distance for anchoring the line posteriorly. In this case, low-voltage tissue at the superior aspect of the inferior vena cava baffle provided the posterior anchor point for the line. Since the AV node is typically located on the pulmonary venous side of the baffle,^3^ this line was also likely to be safe from a heart block point of view. A His signal could not be recorded in this case, but care was taken to look for any change in AV nodal conduction during ablation and for any junctional rhythm. Some slow junctional rhythm was seen with ablation at the posterior aspect of the mitral line, and this led to ablation being ceased and reinitiated intermittently to ensure no impairment to AV nodal conduction.

## Conclusion

To the best of our knowledge, we present the first case of perimitral annular flutter in a patient with a Mustard repair for d-TGA. This was successfully ablated with a septal mitral isthmus line, and the patient has not had any recurrence in 12 months of follow-up.

## Disclosures

The authors have no conflicts of interest to disclose.
